# Chicken or Egg: Is Clonal Hematopoiesis Primarily Caused by Genetic or Epigenetic Aberrations?

**DOI:** 10.3389/fgene.2019.00785

**Published:** 2019-09-10

**Authors:** Olivia Cypris, Tanja Božić, Wolfgang Wagner

**Affiliations:** ^1^Helmholtz-Institute for Biomedical Engineering, Stem Cell Biology and Cellular Engineering, RWTH Aachen University Medical School, Aachen, Germany; ^2^Institute for Biomedical Engineering – Cell Biology, University Hospital of RWTH Aachen, Aachen, Germany

**Keywords:** clonal hematopoiesis, multiple myeloma, epigenetics, epimutation, DNA methylation, aging

## Abstract

Hematopoietic malignancies, including multiple myeloma, are associated with characteristic mutations and genetic instabilities that drive malignant transformation. On the other hand, tumor formation is also associated with drastic epigenetic aberrations, which can impact the genetic sequence. Therefore, the question arises if malignant transformation is primarily caused by genetic or epigenetic events. The tight connection of these processes becomes obvious by the fact that in several malignancies, as well as in age-related clonal hematopoiesis, mutations are particularly observed in epigenetic writers such as *DNMT3A* and *TET2*. On the other hand, specific epigenetic aberrations, so-called “epimutations,” can mimic genomic mutations. In contrast to the genetic sequence, which remains relatively stable throughout life, the epigenome notoriously undergoes drastic changes in normal hematopoietic development and aging. It is conceivable that such epigenetic reorganization, e.g., in 3D chromatin conformation, paves the way for secondary chromosomal instabilities, which then result in tumor-specific genomic changes that further trigger disease progression. This scenario might explain the occurrence of tumor-specific mutations particularly in the elderly. Taken together, the causality dilemma is difficult to solve because genetic and epigenetic aberrations are interlinked during disease development. A better understanding of how the chromatin structure or 3D nuclear organization can evoke specific mutations might provide new perspectives for prevention, early diagnostics, and targeted therapy.

## Malignancies Are Caused by Genomic Aberrations

Genomic instabilities are a hallmark of cancer (Negrini et al., 2010). Already more than a century ago the causal relationship of chromosomal aberrations and dysfunctional mitosis was suggested (Hansemann, 1890; Boveri, 1914), and such research gained significant momentum within the last 20 years with the advent of sequencing technology. For example, in multiple myeloma the relevant genomic aberrations include site-specific mutations, translocations, and gains or losses of parts or whole chromosomes (Morgan et al., 2012; Robiou du Pont et al., 2017). Some of these aberrations have been shown to be of prognostic relevance, such as deletion in 17p13, translocations between chromosome 4 and chromosome 14, or insertions in 1q21, which are rather associated with worse outcome (Fonseca et al., 2009; Neben et al., 2013).

So far, the reasons for the initial genomic instabilities are largely unclear, but it is generally assumed that they simply arise in a stochastic manner. Some passenger mutations may have neutral effects, while others clearly give rise to proliferative advantage, thereby further increasing the risk of malignant transition (Bowman et al., 2018). This process can be accelerated by dysfunctional DNA repair systems as well as impaired chromosome duplication and segregation during mitosis (Difilippantonio et al., 2000; Vargas-Rondon et al., 2017). Furthermore, inhibition of DNA damage response pathway allows cells to proliferate beyond senescence (Greenberg, 2005). Improper chromosome segregation can be caused by telomere shortening, and this may result in chromosome breaks or fusions (Artandi et al., 2000; Hyatt et al., 2017). There is evidence that the order of genomic events is relevant for tumor progression: Initial chromosome translocations can lead to secondary mutations in genes for DNA replication, repair, or genomic stability, which drastically increase occurrence of tertiary genetic aberrations during further development of the disease (Morgan et al., 2012; van Nieuwenhuijzen et al., 2018). In breast cancer, breast cancer 1 (*BRCA1*) mutations often occur after tumor protein 53 (*TP53*) mutations (Martins et al., 2012), because an initial *BRCA1* mutation leads to a cell cycle arrest, which is not in favor of tumor progression (Ashworth et al., 2011). Similarly, the clinical image of myeloproliferative neoplasms was demonstrated to be dependent on the mutation order of ten-eleven translocation 2 (*TET2*) versus janus kinase 2 (*JAK2*): A *JAK2* initial mutation increased the likelihood of presenting with polycythemia vera (as compared to essential thrombocythemia), with an increased risk of thrombosis and an increased sensitivity of *JAK2*-mutant progenitors to ruxolitinib *in vitro* (Ortmann et al., 2015). Taken together, genetic alterations, particularly the mutation order, directly impact the regulation of proliferation, apoptosis, and malignant transformation.

## Epigenetic Alterations in Cancer and Clonal Hematopoiesis

In contrast to genomic changes, epigenetic aberrations do not involve alterations in the DNA sequence. Dynamic modification of DNA and DNA binding proteins plays a crucial role in the regulation of gene expression, chromatin accessibility, and nuclear architecture (Baylin, 2005; Hellman and Chess, 2007; Bocker et al., 2011). Epigenetic marks comprise, for example, DNA methylation and posttranslational modifications of the N-terminal histone tails, such as acetylation, methylation, ubiquitylation, sumoylation, and phosphorylation (Bird, 2002). DNA methylation usually occurs at the fifth carbon atom of a cytosine, particularly in the context of cytosine-guanine (CG) dinucleotides, also referred to as a “CpG site” (Bird, 2002). This process is mediated by DNA methyltransferases (DNMTs), which either maintain existing methylation patterns upon replication (e.g., DNMT1) or create *de novo* patterns (e.g., DNMT3A and DNMT3B) (Okano et al., 1999; Schermelleh et al., 2007). On the other hand, DNA methylation marks can be indirectly removed by TET enzymes, which oxidize 5-methylcytosine into 5-hydroxymethylcytosine. This modification is then either passively depleted upon DNA replication or actively reverted to cytosine by iterative oxidation and thymine DNA glycosylase (TDG)-mediated base excision repair (Kohli and Zhang, 2013).

Cancer cells often reveal genome-wide hypomethylation, which may result from mutations in *DNMTs* or *TETs* (Ko et al., 2010; Russler-Germain et al., 2014). At the same time, tumor-suppressor genes can be silenced by site-specific hypermethylation at promoter regions (Jones and Baylin, 2002). For example, hypermethylation in *TP53*, cyclin-dependent kinase 4 inhibitor B (*CDKN2B*), glutathione peroxidase 3 (*GPX3*), retinol binding protein 1 (*RBP1*), secreted protein acidic and cysteine rich (*SPARC*), and transforming growth factor beta induced (*TGFBI*) was shown to be associated with the transition from the pre-leukemic phase monoclonal gammopathy of undetermined significance (MGUS) to multiple myeloma (Hodge et al., 2005; Kaiser et al., 2013). Furthermore, hypermethylation in multiple myeloma was shown to be enriched in intronic regions associated with B-cell specific enhancer regions (Agirre et al., 2015).

So-called “epimutations” resemble specific epigenetic aberrations that mimic genomic mutations, albeit there is no change in the nucleotide sequence. It has been suggested that such epimutations can contribute in a similar way to malignant transformation as genetic mutations (Peltomaki, 2012; Jost et al., 2014). We have previously demonstrated that acute myeloid leukemia (AML) patients frequently display aberrant hypermethylation in *DNMT3A*, which is rather mutually exclusive with genomic mutations in this gene (Jost et al., 2014). Mutations as well as epimutations in *DNMT3A* seem to be associated with poor prognosis in AML (Jost et al., 2014). Both modifications, mutations and epimutations, may affect alternative splicing of *DNMT3A* (Jost et al., 2014), which is important, because the distinct DNMT3A variants have different effects on the DNA methylation pattern (Božić et al., 2018). In a recent study, we have demonstrated that knockdown and overexpression of specific transcripts of *DNMT3A* has complementary effects on the DNA methylation pattern, gene expression, and differentiation of hematopoietic progenitor cells—thus, alternative splicing of *DNMT3A* has characteristic epigenetic and functional effects (Božić et al., 2018).

Clonal hematopoiesis of indeterminate potential (CHIP) is frequently observed in healthy elderly individuals (Bowman et al., 2018) and may progress into myeloid and lymphoid malignancies (Grossmann et al., 2013; The Cancer Genome Atlas Research Network, 2013). Notably, the mutations that predominantly occur in clonal hematopoiesis are located in the genes *DNMT3A* and *TET2* (Genovese et al., 2014; Xie et al., 2014). These two genes resemble more than 90% of all mutated genes in CHIP (Buscarlet et al., 2017). Overall, mutations in *DNMT3A* are most frequent, whereas *TET2* mutations arise predominantly in older individuals (Kaasinen et al., 2019). Furthermore, mutations in *DNMT3A* and *TET2* are frequently observed in AML (Ley et al., 2010) and to a lesser degree also in multiple myeloma (Dupere-Richer and Licht, 2017). These findings support the notion that modulation of DNA methylation patterns plays a central role in initiation of clonal outgrowth and that mutations in epigenetic writers are early key events in the pathogenesis of hematopoietic malignancies (Corces-Zimmerman et al., 2014; Shlush et al., 2014).

Usually, clones with mutated driver genes have a competitive advantage over their non-mutated counterparts (Abelson et al., 2018). In mice it has been demonstrated that hematopoietic stem cells (HSCs) with loss of *Dnmt3a* reveal enhanced self-renewal and repopulation potential, even after 12 rounds of transplantation, far exceeding that of normal HSCs (Jeong et al., 2018). Mutated HSCs may thus outcompete their native counterparts. While some studies report impaired hematopoiesis (Jeong et al., 2018; Kaasinen et al., 2019), others did not find any significant impact on proliferation or cytopenic effects of either *TET2* or *DNMT3A* mutations in CHIP and found only minor reductions in neutrophils upon *TET2* mutation (Buscarlet et al., 2017). Compared to other driver mutations, *DNMT3A* and *TET2* confer a lower risk of progression to AML, but additional mutations, as shown for example for *Npm1* in mice, can drive CHIP to overt malignant transformation, and such genetic changes make diseases detectable years before diagnosis (Abelson et al., 2018; Loberg et al., 2019). In general, a higher number of mutations and higher variant allele frequencies have a higher risk of AML progression (Abelson et al., 2018). Population dynamics studies in healthy individuals indicated that there are hundreds of thousands of stem cells in the body contributing to hematopoiesis, which divide every 2 to 20 months and on average gain 1.2 mutations per division (Lee-Six et al., 2018). Therefore, branching sub-clones would be expected over many years during clonal evolution of disease progression.

The relevance of epigenetic writers for clonal hematopoiesis and malignant transformation lays the ground for therapeutic regimen that directly impact the epigenetic landscape. Many novel treatment strategies have been developed for multiple myeloma in the past years (Ludwig et al., 2010), and epigenetic regulators resemble promising targets due to the reversibility of epigenetic marks (Alzrigat et al., 2018). Particularly class I and II histone deacetylase (HDAC) inhibitors (such as Varionostat, Panobinostat, and Romidepsin) showed antitumor effects or induced apoptosis *via* the caspase proteolytic pathway (Mimura et al., 2015; Alzrigat et al., 2018). Another promising epigenetic target is the histone methyltransferase enhancer of zeste homolog 2 (EZH2). Inhibition of EZH2 in multiple myeloma cells *in vitro* caused global reduction in H3K27me3 with an antitumor effect in a murine xenograft model (Hernando et al., 2016). DNA demethylating agents, such as 5-azacytidine, are less extensively studied in multiple myeloma as compared to AML. However, there is some evidence that a decrease in global DNA methylation has some anti-myeloma activity, particularly for therapy-resistant cells (Kiziltepe et al., 2007; Khong et al., 2008), and some studies developed biomarkers to estimate the sensitivity of primary myeloma cells for DNMT inhibitors (Moreaux et al., 2012).

## Epigenetic Modifications Can Elicit Genomic Instabilities

Heterochromatin, which is usually highly methylated to maintain its condensed structure, as well as lamina-associated domains, almost never contains actively transcribed genes (van Steensel and Belmont, 2017). The chromatin structure is tightly associated with DNA methylation, since specific enzymes that contain a methyl-CpG-binding domain (MBDs) are able to read CpG methylation and recruit chromatin remodelers, such as HDACs (Espada and Esteller, 2007). Global hypomethylation, which is observed in various types of cancer, may conversely result in loss of heterochromatin and thereby favor gene rearrangements or chromosomal translocations, due to more frequent homologous recombination events (Zhou and Robertson, 2016). Furthermore, global depletion of DNA methylation may affect binding of CCCTC-binding factor (CTCF), which regulates chromatin architecture by mediating distal chromosome interactions (Wang et al., 2012). Chromatin accessibility is additionally controlled by histone modifications (Kouzarides, 2007), and cancer cells particularly display a global loss of histone acetylation and overexpression of histone methyltransferases, such as EZH2 (Fraga et al., 2005; Sharma et al., 2010). Overexpression of EZH2 has been associated with aberrant mitosis and genetic instability in benign mammary epithelial cells and hinders DNA repair through impairment of RAD51 recombinase repair foci formation at sites of DNA breaks (Zeidler et al., 2005; Gonzalez et al., 2011).

**Figure 1:**
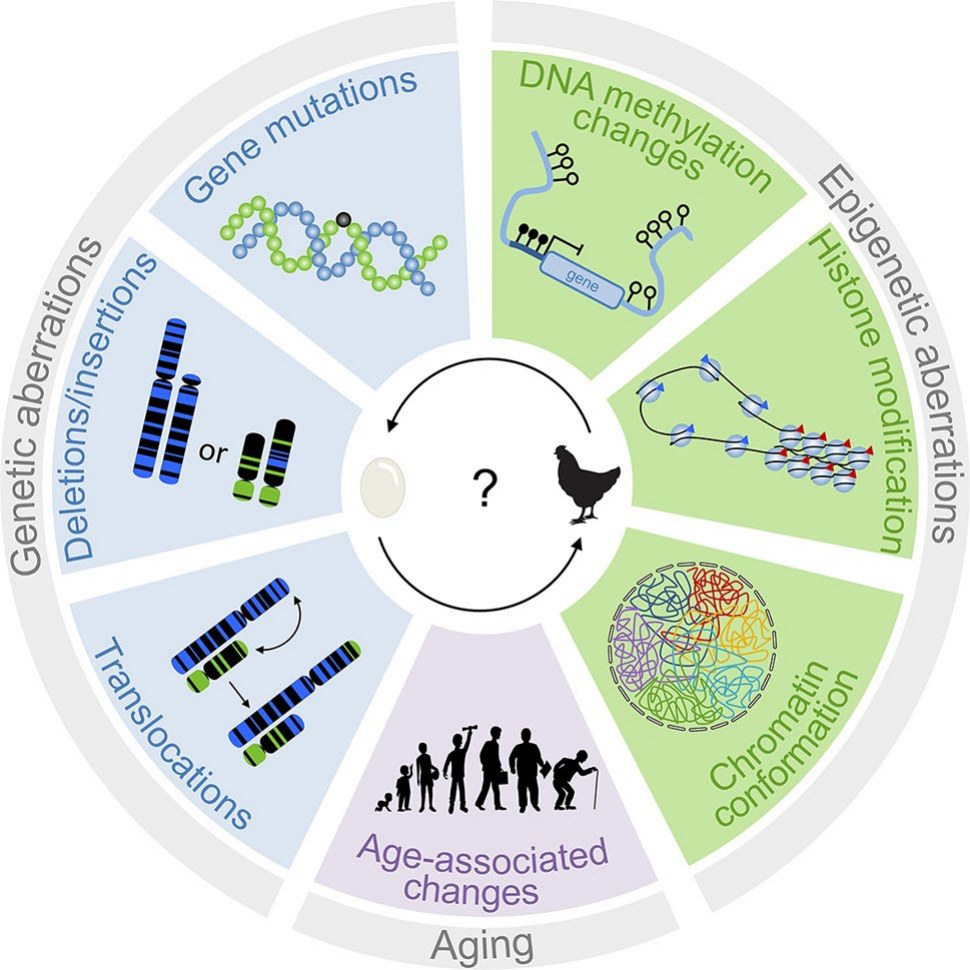
Interplay of genetic and epigenetic aberrations in tumorigenesis. The scheme depicts different genetic and epigenetic aberrations that are relevant for disease development. Genetic changes are traditionally considered to be tumor-initiating events. However, epigenetic changes can also result in genomic instabilities. It is therefore difficult to determine which of these processes is the chicken and the egg.

DNA methylation not only alters chromatin architecture but also influences genomic integrity by stabilizing transposable elements. Hypomethylation in cancer may therefore result in repeat element-directed recombination (Zhou and Robertson, 2016). Treatment of lung cell lines with 5-aza-2′-deoxycytidine activated the expression of retrotransposons, such as long interspersed nuclear element 1 (LINE-1) and Alu elements (Daskalos et al., 2009). In addition, hypomethylation of CpG islands can activate nearby oncogenes (Feinberg and Tycko, 2004). On the other hand, focal hypermethylation can indirectly impact genomic stability by silencing of genes that are relevant for genomic integrity (Brandes et al., 2005) or DNA repair (Esteller et al., 2000; Peng et al., 2006). For example, failure of O^6^-methylguanine repair, e.g., due to hypermethylation in the promotor of the O^6^-methylguanine-DNA methyltransferase (MGMT), results in conversion of G:C to A:T (Esteller et al., 2000). Last but not least, the cytosine methylation itself can act as an endogenous mutagen, because spontaneous deamination of 5-methylcytosine results in conversion to thymine facilitating point mutations, as observed for most hot-spot mutations in *TP53* (Rideout et al., 1990). In fact, it was demonstrated that the occurrence of such methylation-induced point mutations largely differs between cancer types, probably because of varying efficiency of DNA repair mechanisms in those tissues (Sjoblom et al., 2006). Thus, epigenetic modifications play a crucial role for stabilizing genome integrity, and their dysregulation may facilitate genomic instability (**Figure 1**).

## Do Age-Related Epigenetic Changes Trigger Tumorigenesis?

There is a growing perception that aging of the organism is reflected by drastic changes in the epigenetic makeup. Upon aging, there is a global loss of DNA methylation, especially at repetitive elements and transposons, which is also seen in cancer cells (Bollati et al., 2009). Nucleosome occupancy decreases with age (Bochkis et al., 2014), and there is a general decrease in constitutive heterochromatin, which is reflected by a decline of the repressive histone mark H3K9me3 and heterochromatin protein 1 (HP1) (Stewart et al., 2005). Perhaps the most astonishing age-related epigenetic modification is the finding that a large proportion of CpG sites have highly reproducible DNA methylation changes (Weidner and Wagner, 2014). Age-associated DNA methylation changes can be observed across diverse cell types and tissues (Koch and Wagner, 2011). Due to the high reproducibility, age-associated DNA methylation changes can be used to reliably predict the donor age—known as the “epigenetic clock” (Hannum et al., 2013; Horvath, 2013; Weidner et al., 2014). Notably, the rate of epigenetic aging has been linked to life expectancy, indicating that age-associated DNA methylation can also reflect biological aging (Marioni et al., 2015; Lin et al., 2016; Zhang et al., 2017). It is also striking that age-associated DNA methylation patterns are entirely reset upon reprogramming into induced pluripotent stem cells (Frobel et al., 2014; Weidner et al., 2014). However, when it comes to cancer tissue the age predictors fail. In most malignancies the epigenetic clocks are apparently accelerated, whereas they are decelerated in others (Lin and Wagner, 2015). This might be attributed to the fact that tumor tissue recapitulates the epigenetic makeup of the tumor initiating cell, whereas age prediction of healthy tissue is based on a cross section of many cells of the normally developing organism. In fact, there is evidence that age-associated DNA methylation patterns are patient-specific and can be used to track clonal growth (Eipel et al., 2019).

Aging is one of the most relevant risk factors for many types of cancers. Notably, the incidence of cancer diagnosis peaks at different ages for different types of cancer—usually above the age of 50, while, for example, testicular cancer occurs more frequently in younger adults (de Magalhaes, 2013). The reason for this age specificity is not yet fully understood. As indicated above, aging and malignant transformation are to some extent reflected by similar changes in chromatin structure. It is hence conceivable that age-associated epigenetic modifications trigger malignant transformation (Wagner et al., 2015). In fact, epigenetic clocks in cancer, albeit not related to the donor age, correlate with clinical parameters and overall survival in several types of cancer, indicating that regulation of DNA methylation patterns in age-associated CpGs is relevant for cancer development (Lin and Wagner, 2015). Changes in chromatin conformation, which occur commonly at specific ages, might therefore favor tumor-initiating mutations or translocations.

## Future Perspectives

There is clear evidence that genetic as well as epigenetic aberrations contribute to tumor development—the question is as follows: What comes first? Traditionally, the focus is on tumor-specific mutations, which can be easily tracked throughout disease development. On the other hand, malignant transformation is associated with profound epigenetic shifts, which directly impact chromatin conformation and can thereby impact the genetic sequence, as well. A better understanding of how specific epigenetic alterations might favor occurrence of specific genomic lesions will be important. It might then be possible to address such changes for disease prevention, early diagnosis, or directed therapy. A bottleneck for this research is, however, that the available tumor tissue at the time of diagnosis already reflects genome-wide epigenetic aberrations, which makes it difficult to identify the most relevant epigenetic alterations in early stages of malignancy.

## Author Contributions

All authors contributed to writing of this manuscript and reviewed and approved the final version.

## Funding

This work was supported by the Else Kröner-Fresenius-Stiftung (2014_A193), by the Interdisciplinary Center for Clinical Research within the faculty of Medicine at the RWTH Aachen University (O1-3), by the Deutsche Forschungsgemeinschaft (DFG; WA1706/8-1 and WA1706/11-1), by Deutsche Krebshilfe (TRACK-AML), and by the Bundesministerium für Bildung und Forschung (VIP+ Epi-Blood-Count).

## Conflict of Interest Statement

WW is cofounder of Cygenia GmbH (www.cygenia.com), which can provide service for Epigenetic Senescence Signatures and Epigenetic Aging Signatures to other scientists.

The remaining authors declare that the research was conducted in the absence of any commercial or financial relationships that could be construed as a potential conflict of interest.
